# Implementation of real-time energy management strategy based on reinforcement learning for hybrid electric vehicles and simulation validation

**DOI:** 10.1371/journal.pone.0180491

**Published:** 2017-07-03

**Authors:** Zehui Kong, Yuan Zou, Teng Liu

**Affiliations:** National Engineering Laboratory for Electric Vehicles, School of Mechanical Engineering, Institute of Technology, Beijing, China; Chongqing University, CHINA

## Abstract

To further improve the fuel economy of series hybrid electric tracked vehicles, a reinforcement learning (RL)-based real-time energy management strategy is developed in this paper. In order to utilize the statistical characteristics of online driving schedule effectively, a recursive algorithm for the transition probability matrix (TPM) of power-request is derived. The reinforcement learning (RL) is applied to calculate and update the control policy at regular time, adapting to the varying driving conditions. A facing-forward powertrain model is built in detail, including the engine-generator model, battery model and vehicle dynamical model. The robustness and adaptability of real-time energy management strategy are validated through the comparison with the stationary control strategy based on initial transition probability matrix (TPM) generated from a long naturalistic driving cycle in the simulation. Results indicate that proposed method has better fuel economy than stationary one and is more effective in real-time control.

## 1. Introduction

The hybrid electric vehicles (HEVs) are booming rapidly as a solution to the depletion of fossil fuel and severe pollution of air condition. Due to the cooperation of a battery pack and the internal combustion engine, the vehicle powertrain allows the engine to avoid operating at low load with poor efficiency, and the fuel economy and emission can be improved significantly. However, the flexibility of power split also makes energy management problem more challenging.

Energy management strategy (EMS) plays a crucial role in trade-off among performance, fuel economy and emission of HEVs. Numerous studies have been conducted in the energy management of HEVs [1]. Generally, energy management strategies of HEVs are classified into rule-based and optimization-based control strategy [2,3]. The rule-based strategy is widely used in practice due to the straightforward implementation and high computation efficiency. Jalil proposed a rule-based energy management strategy to determine the power split between the engine and battery by setting thresholds [4]. Trovão presented a new rule-based energy management strategy integrating meta-heuristic optimization for a mutilevel EMS in a electric vehicle [5]. To further improve the performance of energy management system, an adaptive fussy logic controller was used to calibrate the operating points and key parameters to minize the fuel consumption according to the driving cycles [6]. However, the performance of any ruel-based strategy is highly dependent on the proper design of the control rules, which usually depends on the enginenering experience. Therefore, many researchers make more efforts to optimization-based energy strategy.

With a prior knowledge of the driving cycles, dynamic programming (DP) receives a optimal result and determines the best fuel economy. However, the real-time and robust performance of this strategy cannot be guaranteed [7]. Instead, DP is implemented offline and served as a benchmark to explore the potential of fuel economy [8]. To make on-line optimization possible, equivalent consumption minimization strategy (ECMS) and model predictive control (MPC) have been adopted to develop energy management [9,10]. ECMS is calculated based on the assumption that the variation of *SOC* (state of charge of the batttery) is neligible due to the slow dynamics compared to other dynamics in HEV [11]. The equivalence factor of ECMS has an important effect on the control performance. However, the optimal value should be determined offline according to a specific cycle [12]. MPC is a promising method for dynamic model due to the prediction ability in a finite future time-horizon. A MPC-based strategy is developed by predicting the road slope. The results show that the method not only maintains the battery *SOC* within its boundary, but also achieves better fuel economy [13]. A Pontryagain’s minimum principle (PMP) is used to find the optimal energy management strategy through combining the power prediction based on the traffic information, such as the maximum acceleration, average velocity and maximum velocity [14]. However, the performance of MPC depends on the prediction accuracy heavily, and varying weather conditions and drving styles make it difficult to guarantee the accuracy.

The exsiting mehods mostly considered a single objective to optimize, such as fuel consuption, while disregarding many onther concerns. The convex multicriteria optimization approach was recently harnessed to optimize mutiple objectives of plug-in hybride electric vehicles, including the battery sizing, charging and on-road power management [15]. Ref. [16] studied the optimal tradeoff between the fuel-cell durability and hydrogen economy for a fuel-cell hybrid bus. Ref. [17] investgates the interactions among three control tasks, such as charging, on-road power management and battery degradation mitigation in samrt grid environment, aiming to minimize the daily operational expense of a PHEV. A high-efficiency convex programming framework is harnessed to minimize daily CO_2_ emissions throgh integrating renewable energy and system-level hybrid powertrain optimization [18].

Several novel algorithms for energy management of HEVs have been developed to realize online optimization for multiple types of HEVs, such as the game theory [19], stochastic dynamic programming (SDP) [20], and reinforcement learning (RL) [21]. Wang optimized the power management problem for a hybrid electric vehicle based on SDP algorithm. However, heavy computation burden makes it difficult to implement online [22]. In numerous areas, RL is a heuristic learning method. Ref. [23] applied RL in the energy management strategy for an electric hybrid tracked vehicle, and compared the performance of RL and SDP. The results indicate that RL algorithm has a better performance and a shorter computation time. However, the RL doesn’t update online and fails to maintain the optimal performance when the driving cycles vary.

This paper proposes a real-time energy management strategy for a small series hybrid electric tracked vehicle. A recursive algorithm is developed to compute the transition probability matrix (TPM) of power-request when the new statistical characteristics of online driving cycle are considerd. RL algorithm is applied to obtain the optimal control policy based on updating TPM of power-request at the regular intervals. A facing-forward simulation model is estabalised to evaluate RL-based real-time energy management strategy through the comparison with RL-stationary control strategy. Simulation results show that the proposed method achieves better fuel economy than RL-stationary one and is feasible for real-time control.

## 2. Modelling of hybrid electric tracked vehicle

### 2.1 Vehicle configuration and parameters

**Fig 1:**
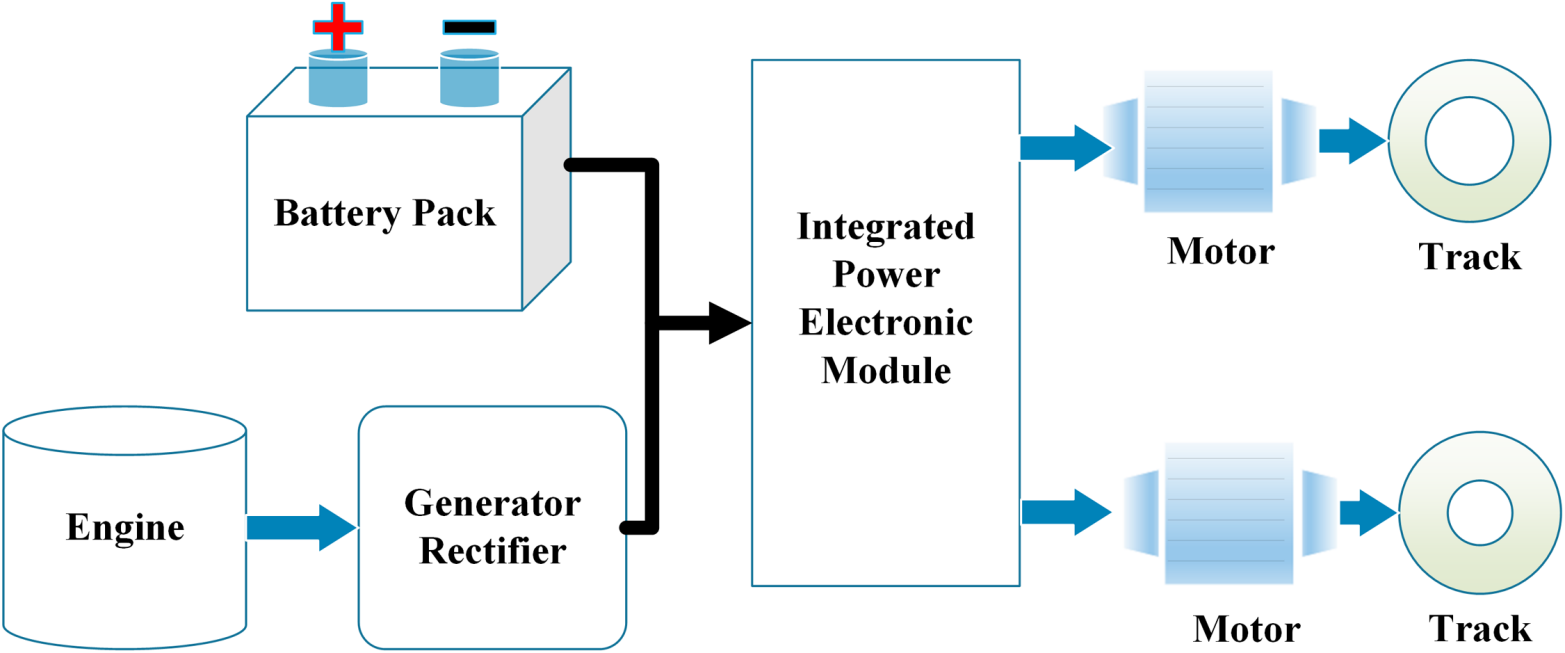
The powertrain structure.

The structure of the hybrid electric tracked vehicle is illustrated in Fig 1. An engine-generator-rectifier set (EGS) and a battery pack supply the electricity to dual motors, propelling the sprocket independently. The engine gives 50 kW maximum power and 93 Nm maximum torque within the speed range from 1200 r/min to 6200 r/min. The generator offers 107 Nm maximum torque within the speed range from 0 r/min to 6400 r/min and 40 kW maximum power. The 37.6 Ah lithium-ion phosphate battery pack gives 307 rated voltage. The essential parameters of the major sub-systems are listed in Table 1.

**Table 1:** Parameters of the major sub-systems.

Symbol	Parameter	Value
*m*	Curb Weight	2500kg
*r*	Sprocket radius	0.15m
*f*	Rolling coefficient	0.05
*A*	Front area	0.7m^2^
*C*_*D*_	Air drag coefficient	0.6
*i*_0_	Ratio from motors to sprockets	41/6
*B*	Tread of the vehicle	1.1m
*K*_*e*_	Electromotive force parameter	0.887V_sard_^-2^
*Kx*	Equivalent resistance coefficient	0.00105NmA^-2^

### 2.2 Vehicle and powertrain modelling

The vehicle is taken as a concentrated mass. The dynamical equation of the vehicle is expressed as

$$ma=F_{TR}-(mg\sin\theta +mgf\cos\theta +C_{D}Av_{ave}{}^{2}/21.15)$$ (1)

where *F*_*TR*_ means the tractive force; *m* is the curb weight, and *a* is the vehicle acceleration; *g* represents the gravity acceleration; *θ* means the road slope angle; *f* is the rolling resistance coefficient; *C*_*D*_ is the aerodynamic drag coefficient; *A* represents the front area of the vehicle; *v*_*ave*_ means the average speed of the two tracks and is determined by *v*_*ave*_ = (*v*_1_+*v*_2_)/2; *v*_1_, *v*_2_ are the speed of the two tracks, respectively.

The demand power to propel the vehicle, denoted by *P*_*dem*_, is calculated by

$$\left\{ \begin{array}{l} P_{dem}=F_{TR}•v_{ave}+M•\omega \\ M=\frac{1}{4}u_{t}mgL\\ u_{t}=u_{\max}\cdot {\left(0.925+0.15R/B\right)}^{-1}\\ R=\frac{v_{ave}}{\left|\omega \right|}\\ \omega =\frac{v_{1}-v_{2}}{B} \end{array}\right.$$ (2)

where *P*_*dem*_ consists of two parts, straight power and steering power. *M* is the resisting yaw moment; *ω* represents the rotational speed; *u*_*t*_ is the lateral resistance coefficient; *R* is the turning radius; *L* is behalf of the contacting track width, and *B* means the tread of the vehicle; *u*_*max*_ is the maximum steering resistance coefficient with the radius of braking steering, *R* = *B*/2.

**Fig 2:**
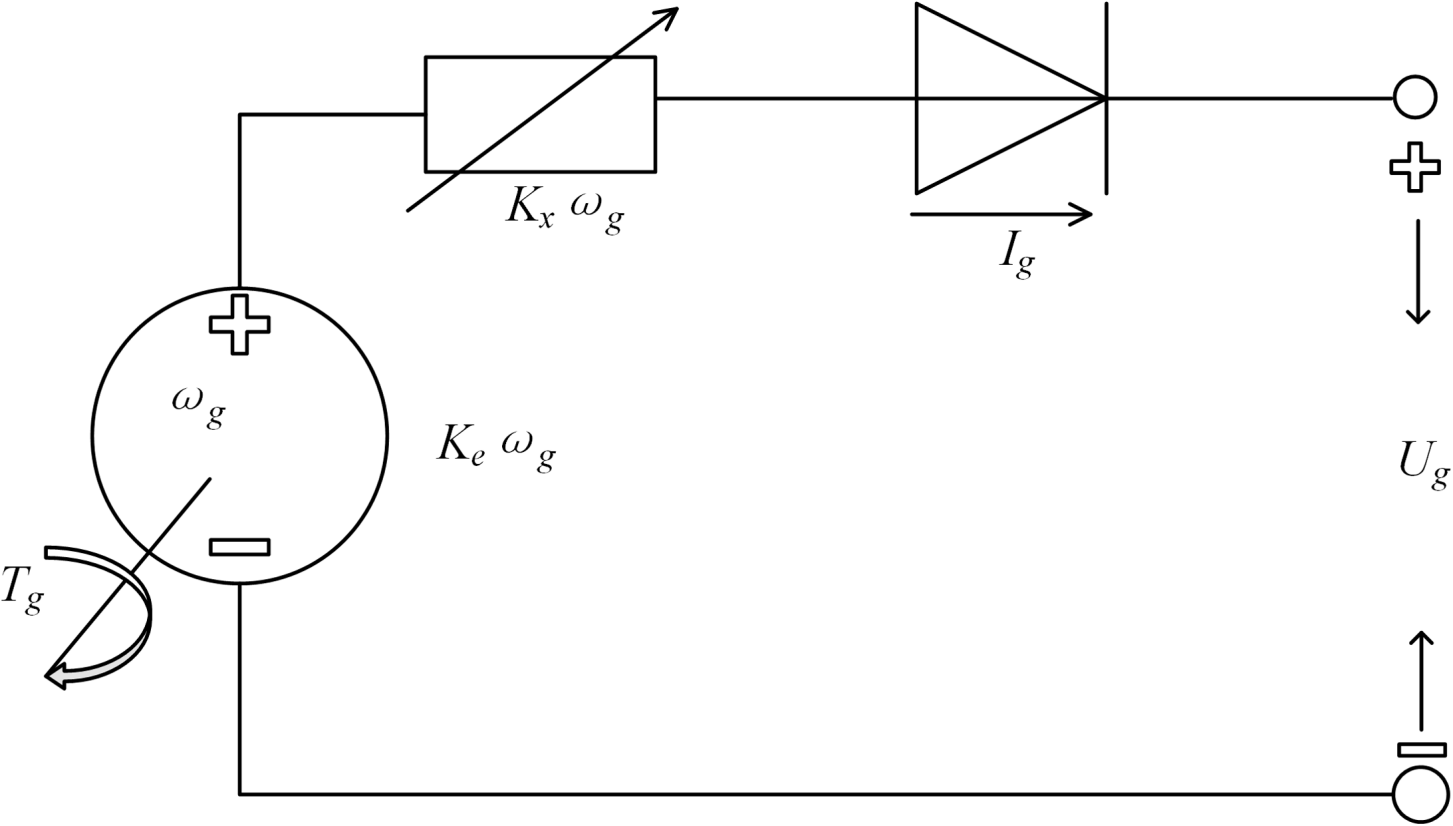
Equivalent electric circuit of the EGS.

To analyze and evaluate the EGS’s fuel economy, the equivalent electric circuit is established in Fig 2, which consists of the diesel engine, permanent magnet generator, and rectifier. The output voltage and electromagnetic torque, *U*_*g*_ and *T*_*g*_ of the generator are determined as [24]

$$\left\{ \begin{array}{l} U_{g}=K_{e}\omega_{g}-K_{x}\omega_{g}I_{g}\\ T_{g}=K_{e}I_{g}-K_{x}I_{g}^{2} \end{array}\right.$$ (3)

where *ω*_*g*_ is the angular velocity; *K*_*e*_*ω*_*g*_ represents the electromotive force; *K*_*x*_ is the equivalent resistance coefficient and *K*_*x*_ = 3*PL*^*g*^/*π*; *L*^*g*^ is the synchronous inductance of the armature, and *P* is the number of poles; *I*_*g*_ and *U*_*g*_ are the current and output voltage of the generator.

The fuel consumption of the vehicle is determined by the engine torque *T*_*eng*_ and speed *n*_*eng*_. The speed of the engine is determined as follows.

$$\left\{ \begin{array}{l} (J_{\text{eng}}+J_{\text{g}})\frac{\pi }{30}\frac{dn_{\text{g}}}{dt}=T_{\text{eng}}-T_{\text{g}}\\ n_{eng}=n_{g} \end{array}\right.$$ (4)

where *n*_*g*_, *n*_*eng*_ denote the rotational speed of the generator and engine, respectively; *J*_eng_ and *J*_g_ are the moments of inertia for the engine and generator; *T*_*eng*_ is the torque of engine and *T*_*g*_ is the electromagnetic torque of the generator.

An internal-resistance model is used to reflect *SOC* dynamics as [25]

$$\left\{ \begin{array}{l} \frac{d\text{SOC}}{dt}=-\frac{I_{b}}{C_{b}}\\ SOC_{\min}\leq SOC(t)\leq SOC_{m\text{ax}} \end{array}\right.$$ (5)

where *C*_*b*_ is the battery capacity; *I*_*b*_ means the battery current which is calculated by

$$\left\{ \begin{array}{l} I_{b}=\frac{\left(V_{OC}-\sqrt{V_{OC}{}^{2}-4R_{int}P_{b}}\right)}{2R_{int}}\\ I_{b,\min}\leq I_{b}(t)\leq I_{b,\max} \end{array}\right.$$ (6)

where *V*_*OC*_ is the open circuit voltage; *R*_*int*_ means the internal resistance of battery and *P*_*b*_ is the output power of the battery.

### 2.3 Optimal energy management problem formulation

Cost function is the trade-off of the fuel consumption and *SOC* variation, ensuring that the final *SOC* of battery stays at the same level of the initial value, expressed as follows

$$J=\int_{t_{0}}^{t_{f}}(\overset{\cdot }{fuel}(t)+\beta {\left(SOC(t)-SOC_{ref}\right)}^{2})dt$$ (7)

where *β* represents the penalty factor, which is a positive weighting factor; *fuel* is the fuel consumption at time *t*. The rate of flowing fuel mass $\overset{\cdot }{fuel}(t)$ is determined by engine torque *T*_*eng*_ and rotational speed *n*_*eng*_ based on BSFC (braked specific fuel consumption) map, typically obtained through a bench test. The engine’s torque is used to regulate the power split between the EGS and battery to minimize the total fuel consumption.

## 3. Real-time energy management strategy

### 3.1 Online updating transition probability matrix (TPM)

The power-request of the vehicle is calculated according to the Eq (2). Using the maximum likelihood estimation and nearest method, the transition probability matrix of the power-request is described as

$$p_{ij}=\frac{N_{ij}}{N_{oi}}=\frac{N_{ij}(k)/k}{N_{oi}(k)/k}=\frac{F_{ij}(k)}{F_{oi}(k)}$$ (8)

where *N*_*ij*_ denotes the transition numbers from state *x*_*i*_ to state *x*_*j*_, and *N*_*oi*_ is the total transition numbers initiated from state *x*_*i*_; *k* means the number of transition; *F*_*ij*_ is the total frequency rate of transition event *f*_*ij*_ from state *x*_*i*_ to state *x*_*j*_ and *F*_*oi*_ is the frequency rate of transition event *f*_*i*_ initiated from state *x*_*i*_. *f*_*ij*_(*t*) = 1 if a transition is occurred from *x*_*i*_ to *x*_*j*_ at time instant *t*; *f*_*i*_(*t*) = 1 if there occurs a transition initiated from *x*_*i*_ at time instant *t*, otherwise, these take zero values. Then $\sum_{t=1}^{k}f_{ij}(t)$ = *N*_*ij*_(*k*) and $\sum_{t=1}^{k}f_{i}(t)$ = *N*_*i*_(*k*). The frequency rate *F*_*ij*_ and *F*_*i*_ are calculated as follows:

$$\left\{ \begin{array}{l} F_{ij}(k)=\frac{N_{ij}(k)}{k}=\frac{1}{k}\sum_{t=1}^{k}f_{ij}(t)\\ F_{oi}(k)=\frac{N_{oi}(k)}{k}=\frac{1}{k}\sum_{t=1}^{k}f_{i}(t) \end{array}\right.$$ (9)

and the recursive expressions are deduced [26].

$$\left\{ \begin{array}{l} F_{ij}(k)=\frac{1}{k}\sum_{t=1}^{k}f_{ij}(t)=\frac{1}{k}[(k-1)F_{ij}(k-1)+f_{ij}(k)]\\ \;=F_{ij}(k-1)+\frac{1}{k}[f_{ij}(k)-F_{ij}(k-1)]\\ F_{oi}(k)=\frac{1}{k}\sum_{t=1}^{k}f_{i}(t)=\frac{1}{k}[(k-1)F_{oi}(k-1)+f_{i}(k)]\\ \;=F_{oi}(k-1)+\frac{1}{k}[f_{i}(k)-F_{oi}(k-1)] \end{array}\right.$$ (10)

where 1/*k* is replaced by a constant *ψ* ranging from 0 to 1, namely forgetting factor to determine the effective memory depth of historic driving cycle, and the forgetting factor *ψ* is set to be 0.01 in this paper.

By substituting the expression (10) into (8), the recursive algorithm of TPM is derived for online learning as follows:

$$p_{ij}=\frac{F_{ij}(k)}{F_{oi}(k)}=\frac{F_{ij}(k-1)+\psi [f_{ij}(k)-F_{ij}(k-1)]}{F_{oi}(k-1)+\psi [f_{i}(k)-F_{oi}(k-1)]}$$ (11)

### 3.2 RL-based real-time energy management strategy

The driving schedule is considered as a finite Markov decision process, which comprises a set of state variables *s*_*t*_∈*S* = {*SOC*(*t*),*n*_*g*_ (*t*)|0.6≤*SOC*(*t*)≤0.9, 0≤*n*_*g*_ (*t*)≤6400}, a set of actions *a*_*t*_∈*A* = {*T*_*eng*_ (*t*)|0≤*T*_*eng*_ (*t*)≤93}, a reward function *r*_*t*_ ∈*Reward* = {*fuel*(*s*_*t*_, *a*_*t*_)}.

Corresponding to the state *s* and action *a*, the optimal value of the state *s*_*t*_ is defined as the expected finite discounted sum of the rewards, as follows [27]:

$$\begin{array}{l} V^{*}(s_{t})=\underset{\pi }{\min}E(\sum_{t=t_{0}}^{\;t=t_{f}}\gamma^{t}r_{t})\\ \;=\underset{a_{t}}{\min}(r_{t}(s_{t},a_{t})+\gamma \sum_{s_{t}^{'}\in S}p_{s_{t}a_{t},s_{t}^{\prime }}V^{*}(s_{t}^{\prime }))\;\forall s_{t}\in S \end{array}$$ (12)

where *π* is a control policy, *γ*∈[0, 1] is a discount factor; *p* means the probability of the occurrence of a transition from state *s*_*t*_ to *s*_*t*_*’* under action *a*_*t*_. And the optimal control policy can be decided by the function

$$\begin{array}{l} \pi^{*}(s_{t})=\arg\;\underset{a_{t}}{\min}\;E(\sum_{t=t_{0}}^{\;t=t_{f}}\gamma^{t}r_{t})\\ \;=\arg\;\underset{a_{t}}{\min}(r_{t}(s_{t},a_{t})+\gamma \sum_{s_{t}^{'}\in S}p_{s_{t}a_{t},s_{t}^{\prime }}V^{*}(s_{t}^{\prime })) \end{array}$$ (13)

In addition, the *Q* value *Q*(*s*_*t*_, *a*_*t*_) and optimal value Q^*^ corresponding to state *s*_*t*_ and action *a*_*t*_ are defined as follows

$$\left\{ \begin{array}{l} Q(s_{t},a_{t})=r_{t}(s_{t},a_{t})+\gamma \sum_{s_{t}^{\prime }\in S}p_{s_{t}a_{t},s_{t}^{\prime }}Q(s_{t}^{\prime },a_{t}^{\prime })\left(s^{\prime },a_{t}^{\prime }\right)\\ Q^{*}(s_{t},a_{t})=r_{t}(s_{t},a_{t})+\gamma \sum_{s_{t}^{\prime }\in S}p_{s_{t}a_{t},s_{t}^{\prime }}\underset{a^{'}}{\min}Q^{*}(s_{t}^{\prime },a_{t}^{\prime }) \end{array}\right.$$ (14)

**Fig 3:**
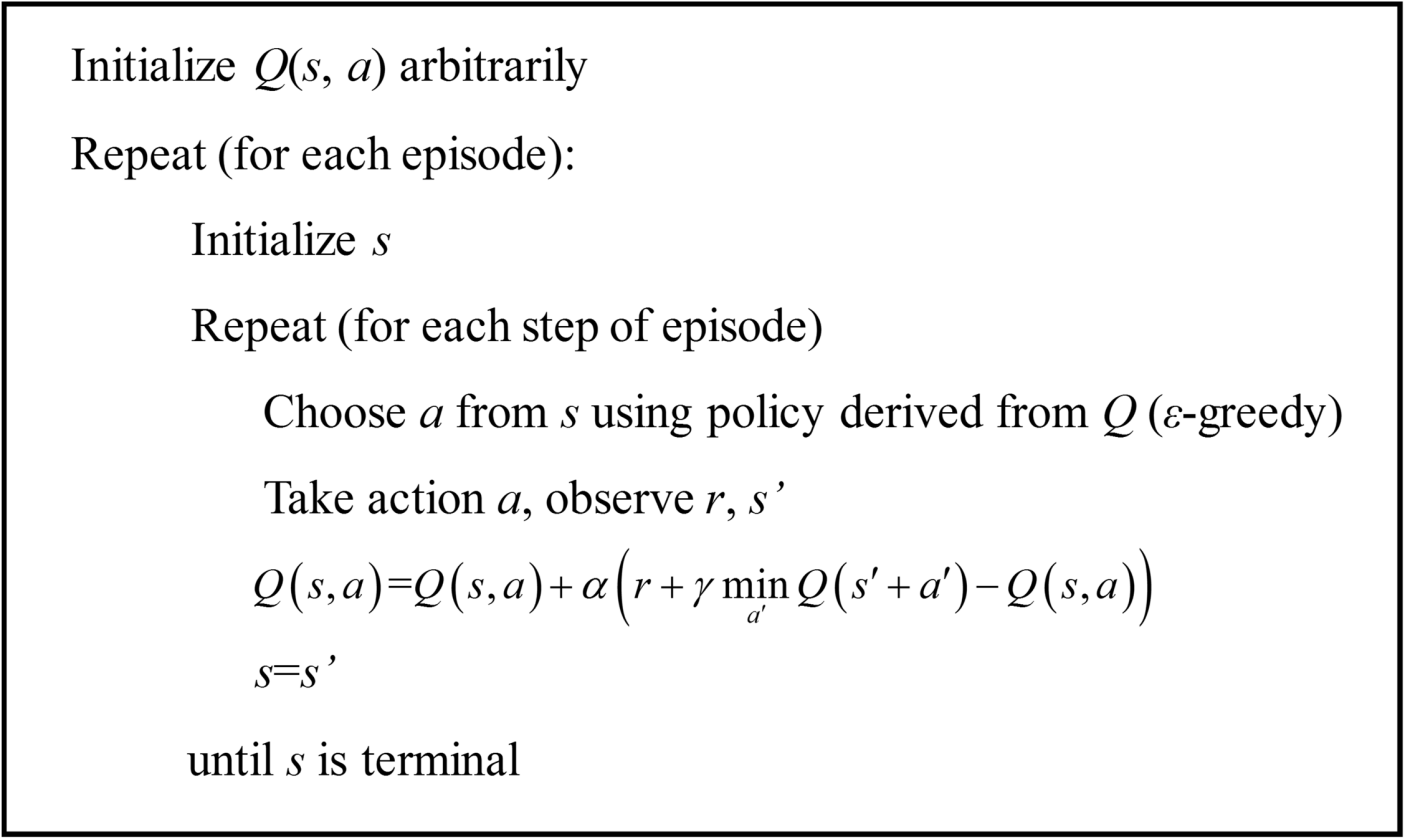
The pseud code of *Q*-learning.

Finally, the updating rule of *Q*-learning and optimal control strategy are defined as [28, 29]:

$$\left\{ \begin{array}{l} Q\left(s_{t},a_{t}\right)≔Q\left(s_{t},a_{t}\right)+\alpha \left(r_{t}+\gamma \underset{a^{\prime }}{\min}Q\left(s_{t}^{\prime }+a_{t}^{\prime }\right)-Q\left(s_{t},a_{t}\right)\right)\\ \pi^{*}(s_{t})=\arg\;\underset{a_{t}}{\min}\;Q^{*}(s_{t},a_{t}) \end{array}\right.$$ (15)

where *γ*∈[0, 1], *α*∈[0, 1] are respectively discount factor and decayed factor in *Q*-learning. The computational flowchart of RL is listed in the pseud code format as shown in Fig 3.

**Fig 4:**
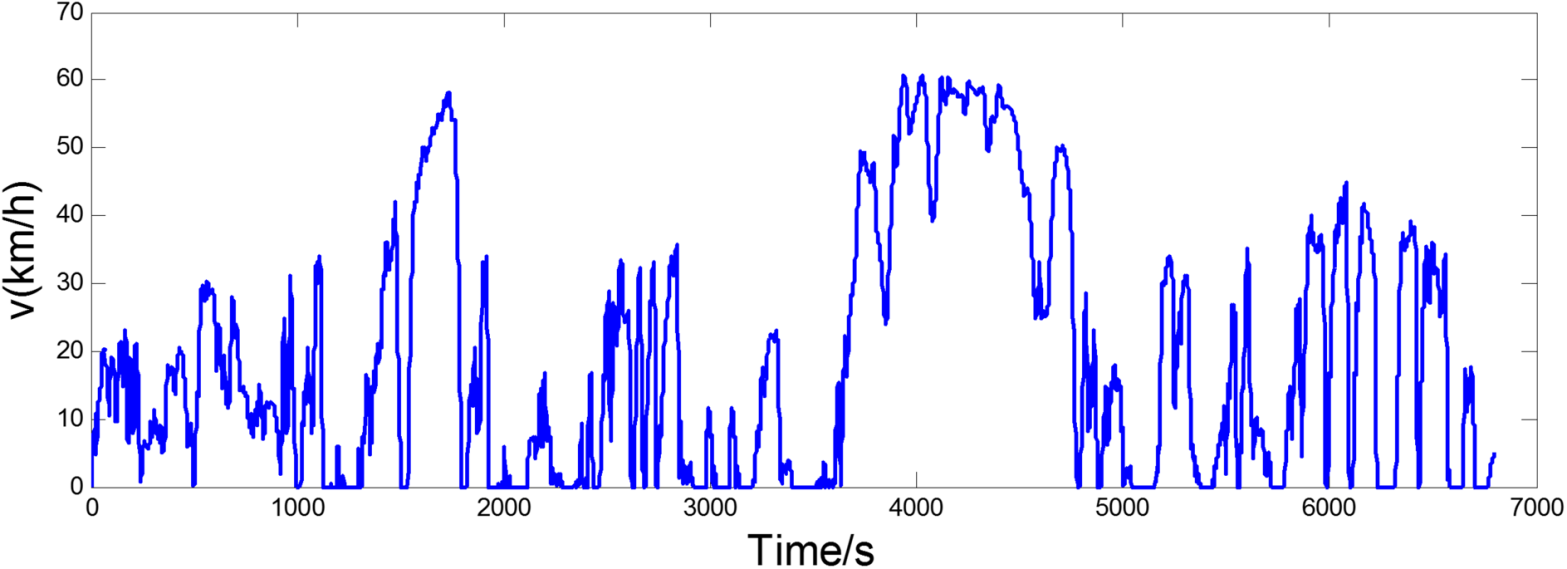
A long naturalistic driving cycle.

**Fig 5:**
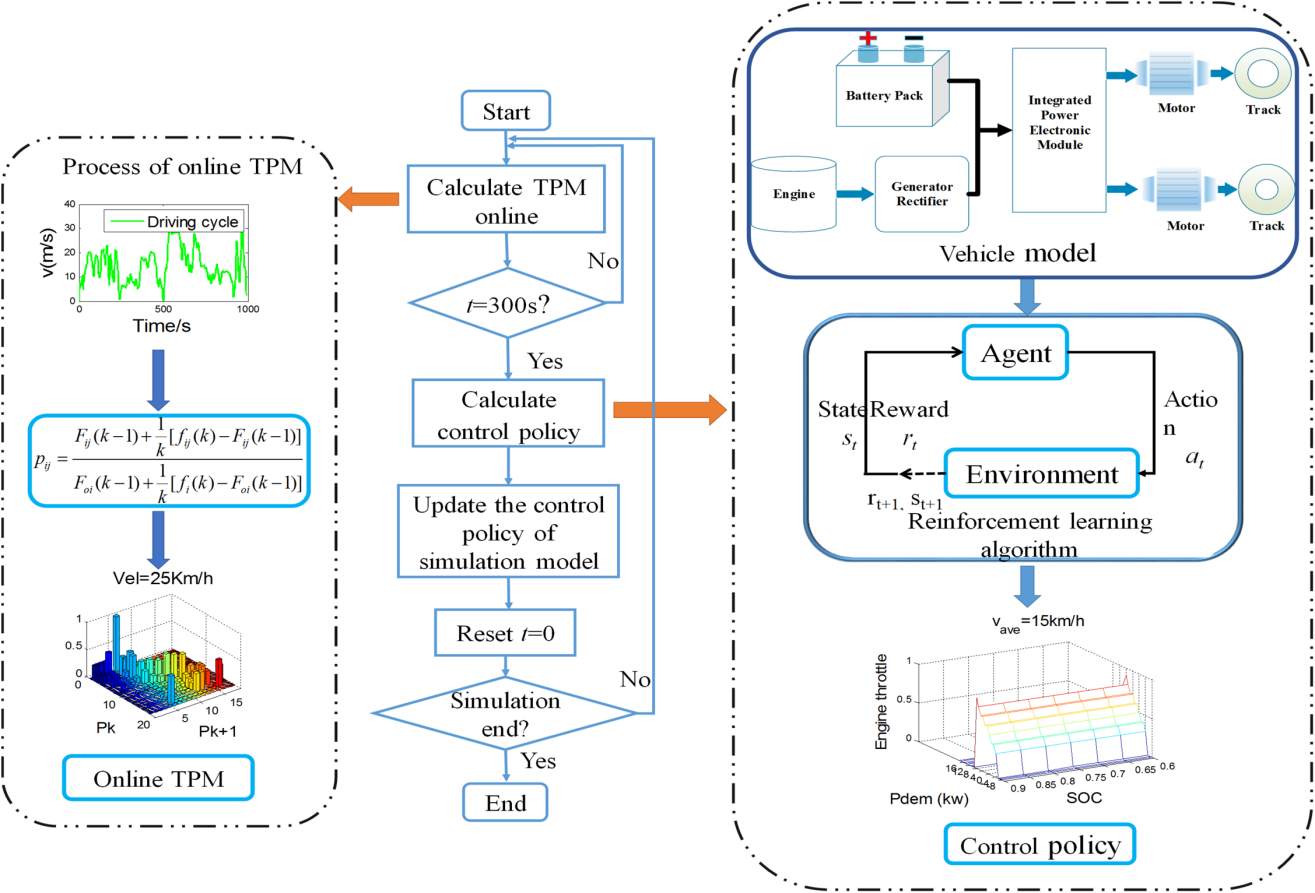
Process of real-time energy management strategy design.

Through adopting *Q*-learning algorithm above, the stationary control policy is derived based on initial TPM generated from a long naturalistic driving cycle as shown in Fig 4. This paper proposes a real-time energy management strategy, aiming at improving the adaptability to change of power-request characteristics. Firstly, the updating TPM of power-request is acquired according to Eq (11) in real-time. Then at set intervals, the control policy is recalculated by using *Q*-learning algorithm, adapting the controller to the varying driving schedules. Table 2 lists the fuel consumption and calculation time in cases of choosing different time interval. The results show that the fuel consumptions have no big difference, with 50 s, 100 s, 200 s and 300 s selected as the time interval. When fuel consumptions are similar, the case of longer time interval can reduce the updating number of control policy and calculation time. Therefore, the time interval of updating control policy is set to be 300 s in this paper. And the design process of the real-time energy management strategy is depicted in Fig 5.

**Table 2:** Fuel consumption.

Time interval (s)	Fuel consumption(g)	Relative increase	Calculation time (h)	Updating number
50	430.2	—	9.5	19
100	430.8	0.1%	4.5	9
200	432.0	0.4%	2	4
300	432.5	0.5%	1.5	3
400	445.8	3.6%	1	2

## 4. Simulation and validation

**Fig 6:**
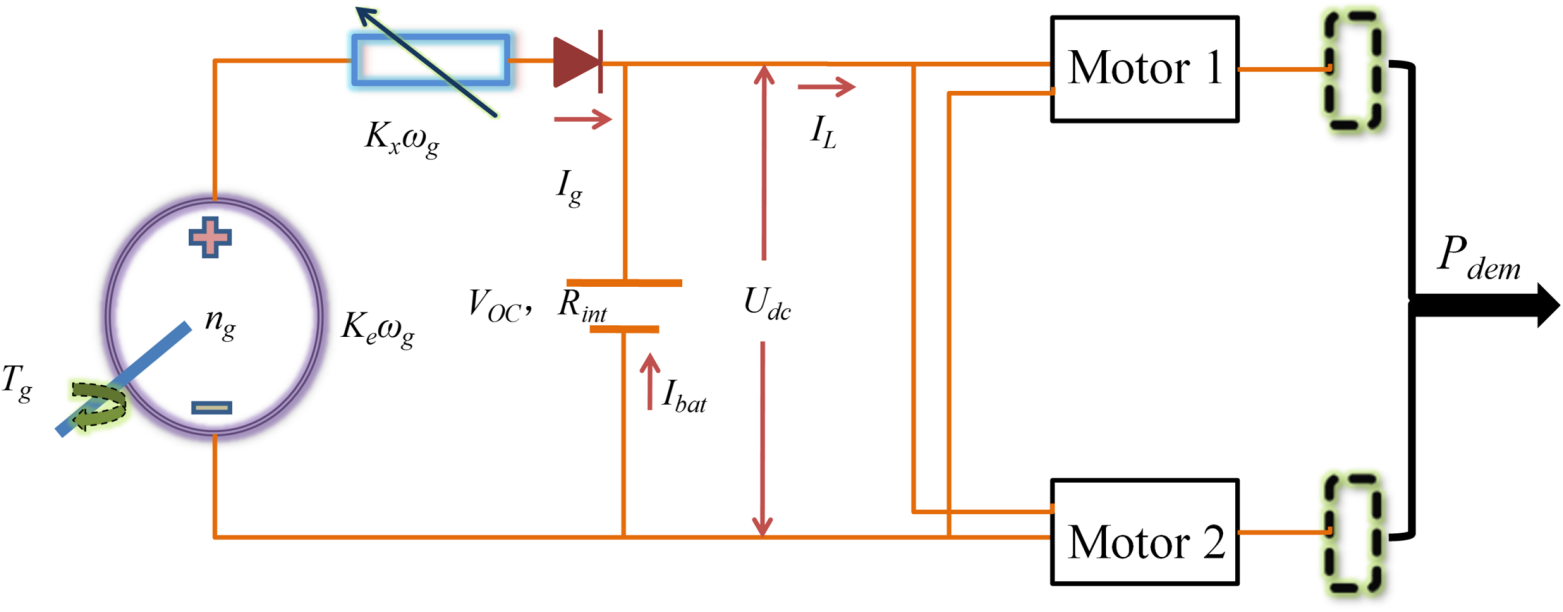
The simplified electric coupling model.

**Fig 7:**
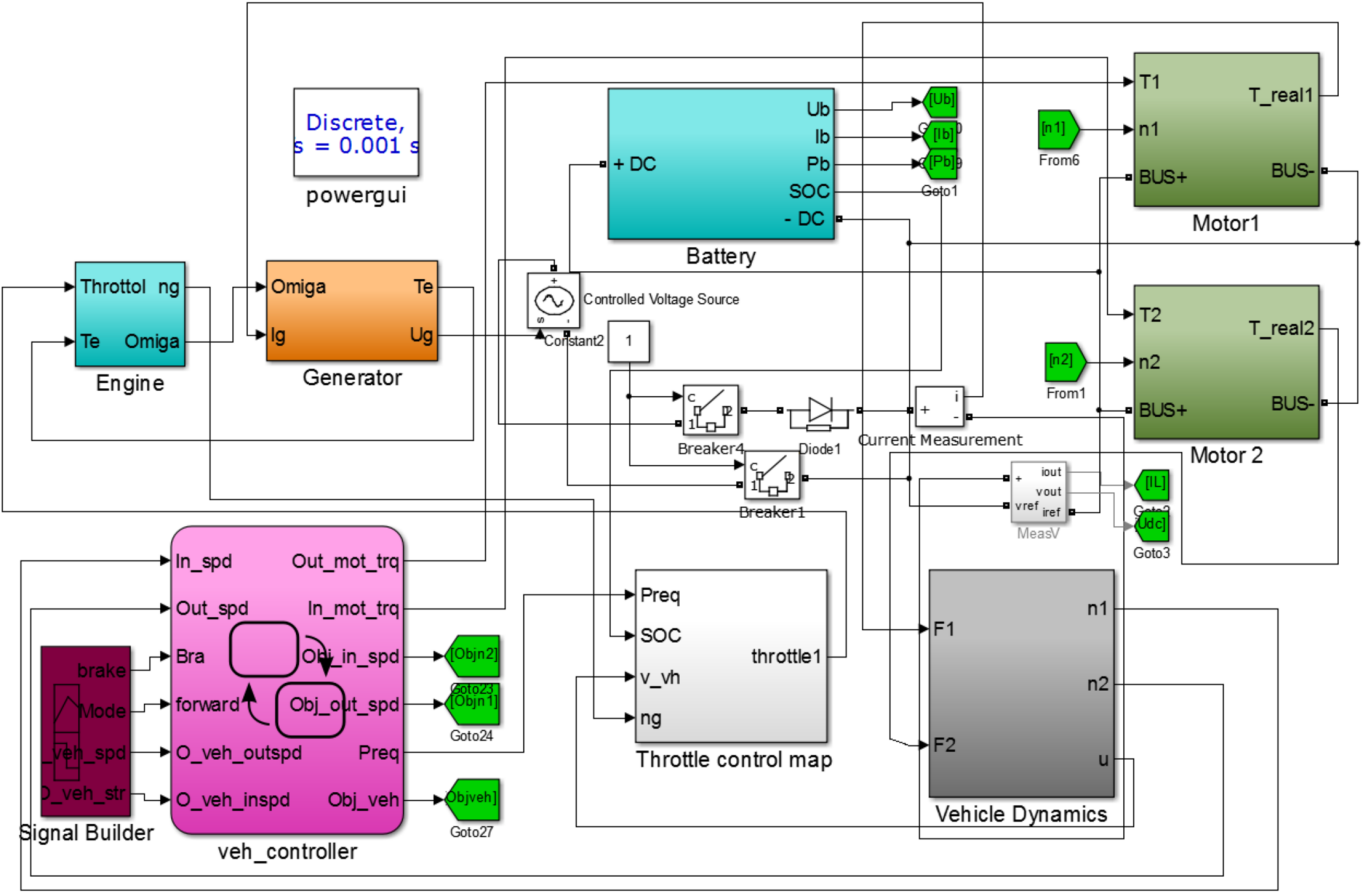
The facing-forward simulation model.

The simplified electric coupling model of powertrain for hybrid electric tracked vehicle is given in Fig 6, where the power provided from EGS and battery should keep balance with the power-request of two driving motors. Based on the electric coupling model and powertrain model in Section 2, a facing-forward detailed model for the hybrid electric tracked vehicle is established in the Simulink environment, consisting of the engine-generator model, power battery pack, two motors, vehicle dynamic model and controller as shown in Fig 7. The proportional-integral driver model is adopted to adjust the toques of both motors to follow the target driving cycles. The controller determines the throttle according to the control map obtained through RL algorithm when the states of vehicle feedbacks to the controller. The RL-based real-time energy management strategy and stationary energy management strategy are applied into the facing-forward simulation model for the same driving cycle, respectively. The control map remains unchanged in the case of stationary strategy while the control map is updated every 300s in the case of real-time strategy. The initial values of the state variables *n*_*g*_ and *SOC* are taken as 1200 rpm and 0.75, respectively.

**Fig 8:**
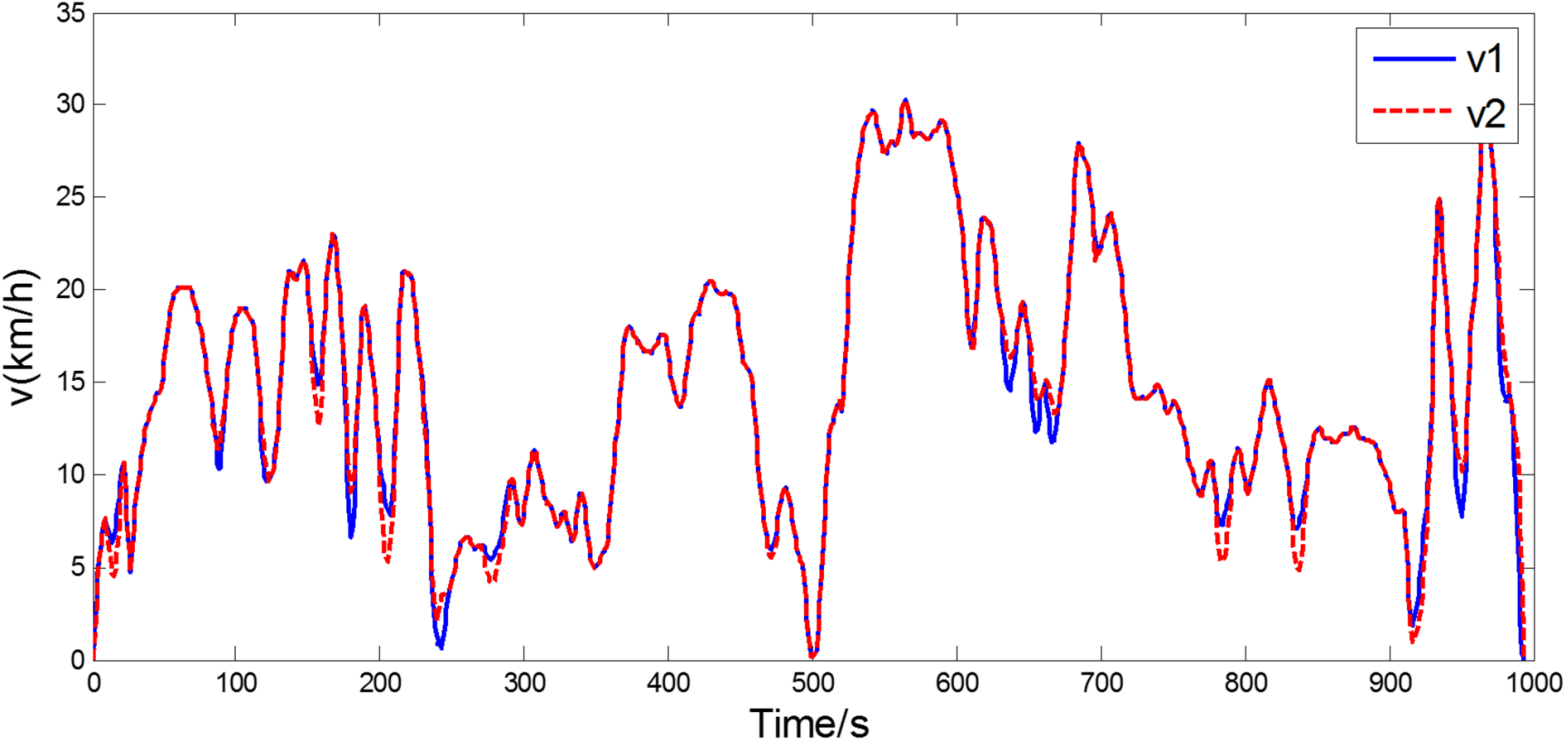
The experimental driving schedule.

**Fig 9:**
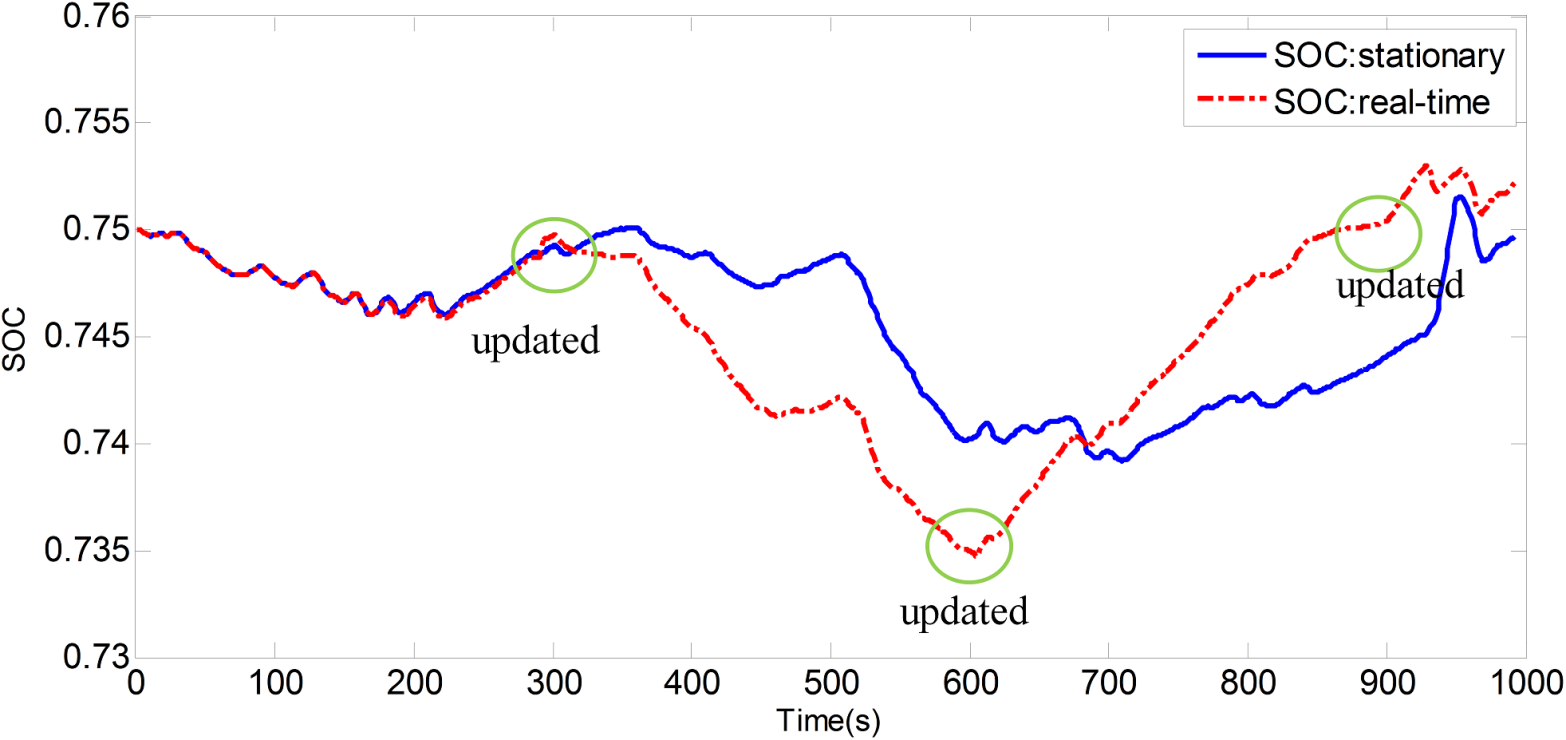
*SOC* trajectory in different strategies.

**Fig 10:**
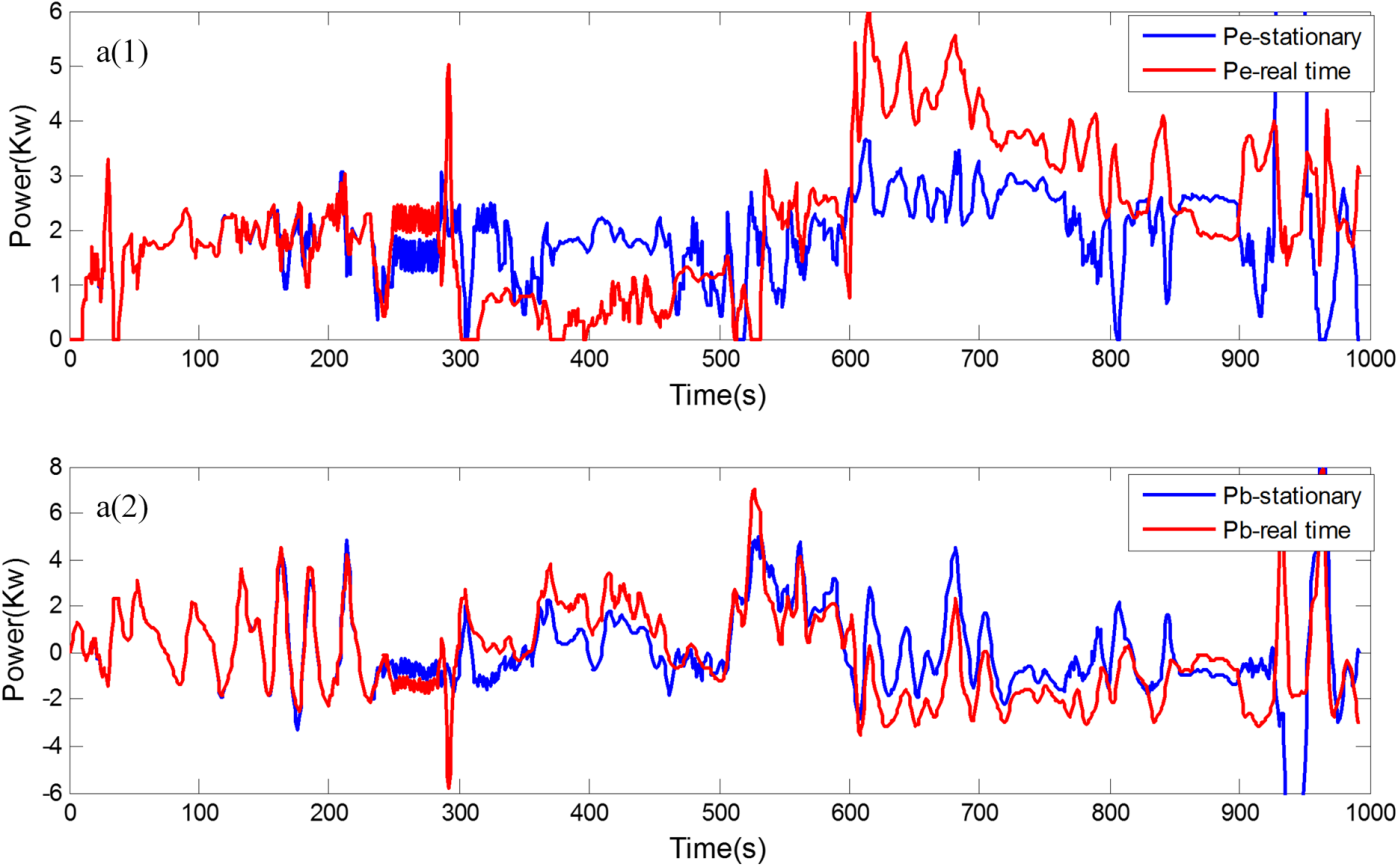
Power split. a1 is power of the engine, and a2 is the power of the battery.

**Fig 11:**
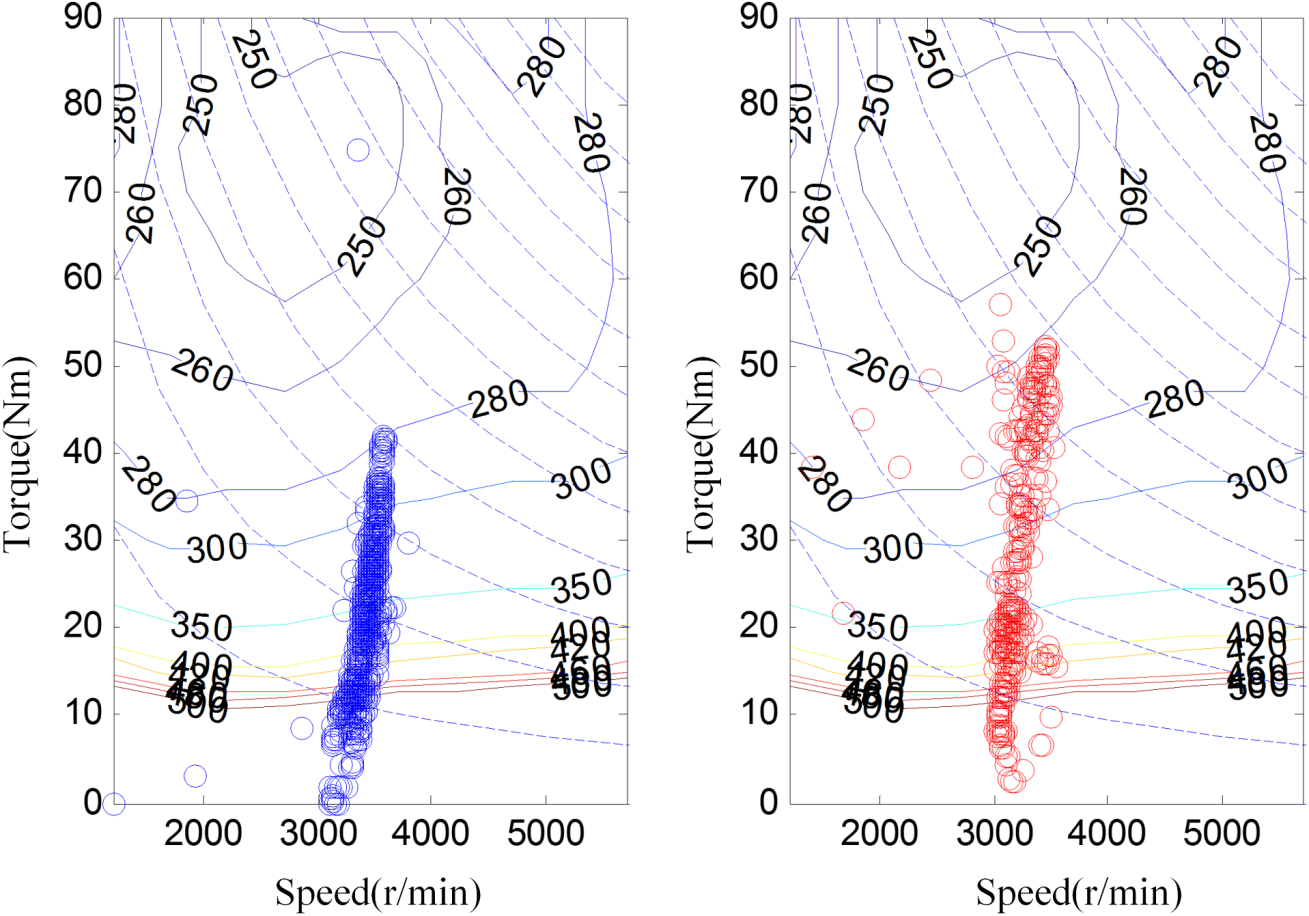
Working points of the engine.

Fig 8 shows the experimental driving schedule used in the simulation to validate the proposed method. The *SOC* trajectories and power split between EGS and battery for the experimental driving schedule are illustrated in Fig 9 and Fig 10. Final *SOC* of two methods were close to initial *SOC* value due to the final constraints for *SOC* value. During the first 300 seconds, two control strategies are based on the same TPM, so *SOC* trajectories change almost in the same way. However, when the driving condition changes, the control policy is recalculated at 300 s for real-time control strategy. And the same process is triggered at 600 s and 900 s. Similarly, the power split changes correspondingly as shown in Fig 10. For the sake of eliminating the influence of the deviation between the final *SOC* on fuel consumption, an *SOC*-correction method [30] is utilized to compensate for the fuel consumption. Table 3 shows the fuel consumption of two methods after *SOC* correction. The fuel consumption of RL-based real-time strategy is 6% lower than that of stationary strategy. Fig 11 depicts the working points of the engine for the experimental driving schedule. There are more points in the optimal fuel consumption area in the real-time strategy than that of stationary one.

**Table 3:** Fuel consumption for the experimental driving schedule.

Algorithms	Fuel consumption(g)	Relative increase	Final SOC
RL online	430.856	—	0.7497
Stationary	456.707	6%	0.7522

**Fig 12:**
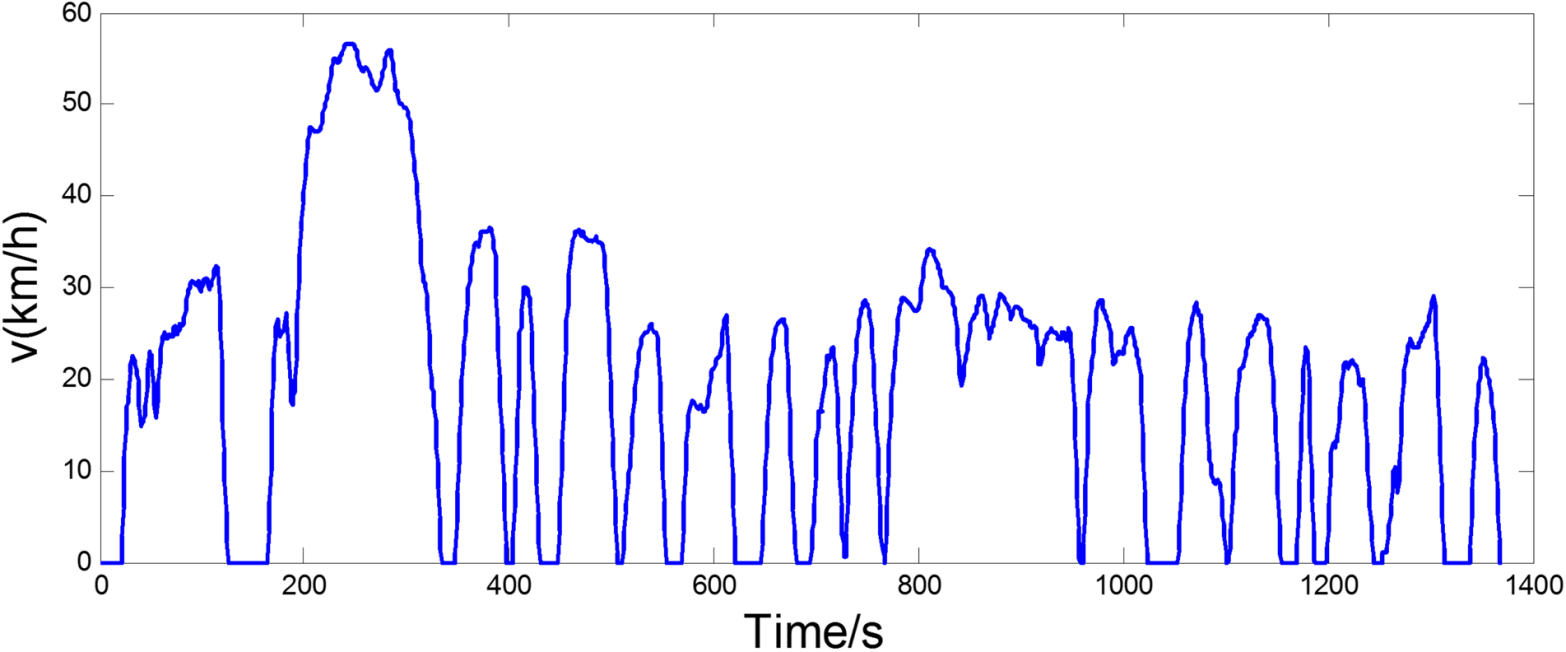
The validation driving schedule.

**Fig 13:**
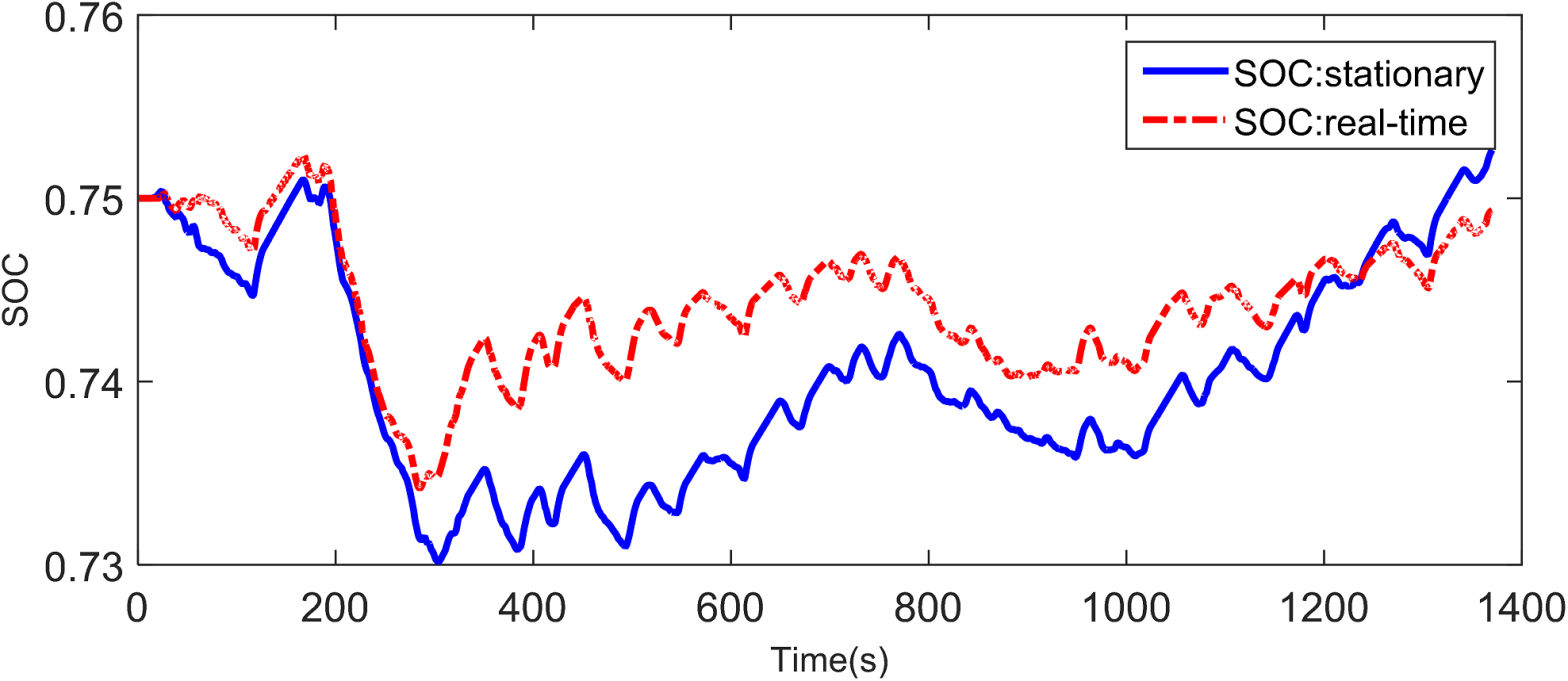
SOC trajectories for the validation driving schedule.

**Fig 14:**
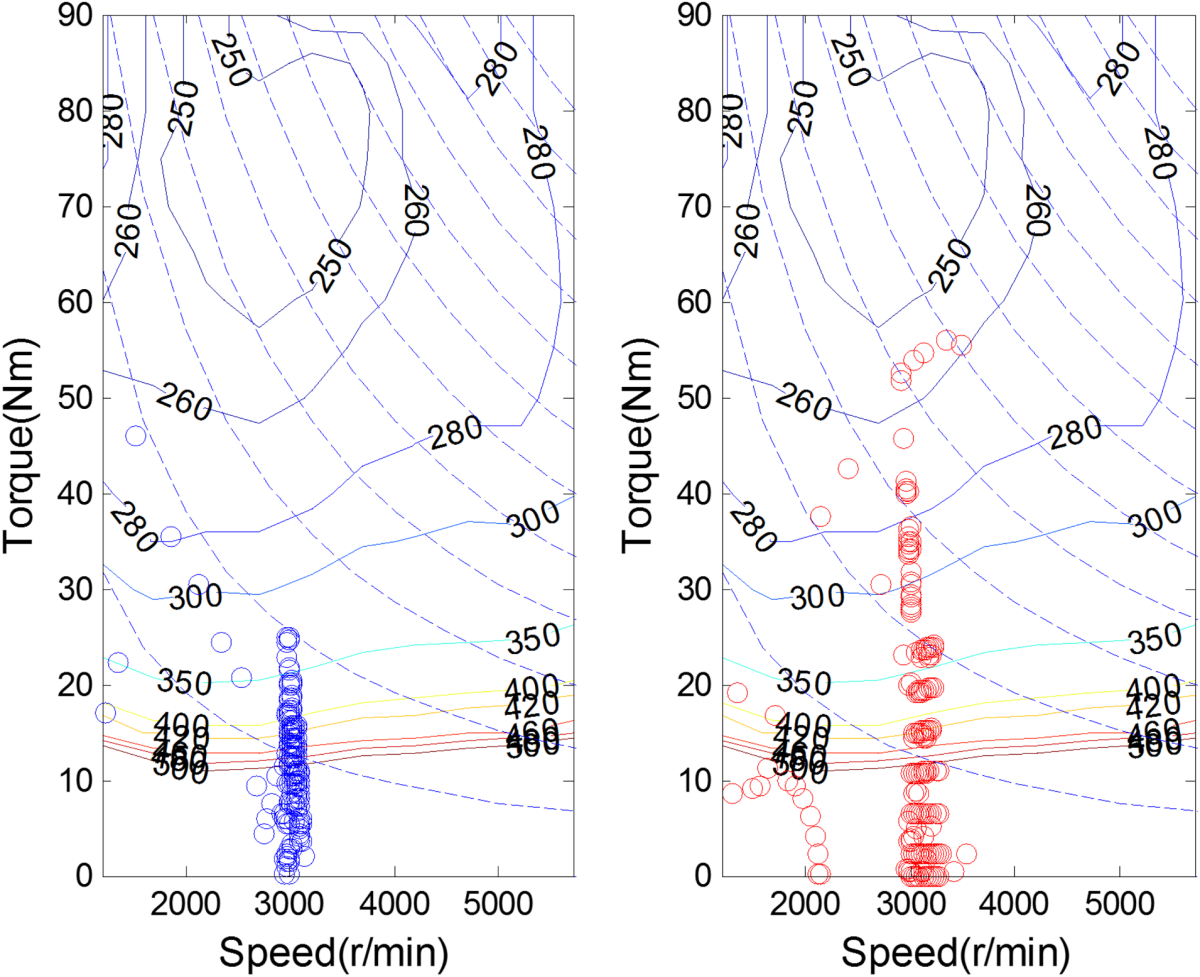
Working points of the engine.

To further explore the adaptability and robustness of RL-based real-time strategy, the same procedure is performed for the validation driving schedule in the field test (shown in Fig 12). The *SOC* trajectories and working points of engine are shown in Fig 13 and Fig 14. Because of the constraint for *SOC* value, the final *SOC* values are close to the initial *SOC* value. Table 4 lists the fuel consumption after *SOC* correction. Due to utilizing the newest characteristics of driving schedule, the RL-based real-time control strategy can reduce about 8% fuel consumption than stationary one. The reason why the fuel improvement is bigger in the later simulation than the former is that the driving condition during the whole validation driving schedule is similar, as presented in Fig 12, and the control policy based on updating TPM at 600 s has already included the statistical information of whole driving schedule. The results suggest that the real-time control strategy has a good performance of robustness and enable to adapt to different driving cycles better.

**Table 4:** Fuel consumption for the validation driving schedule.

Algorithms	Fuel consumption(g)	Relative increase	Final SOC
RL online	584.275	—	0.7526
Stationary	635.865	8%	0.7493

**Fig 15:**
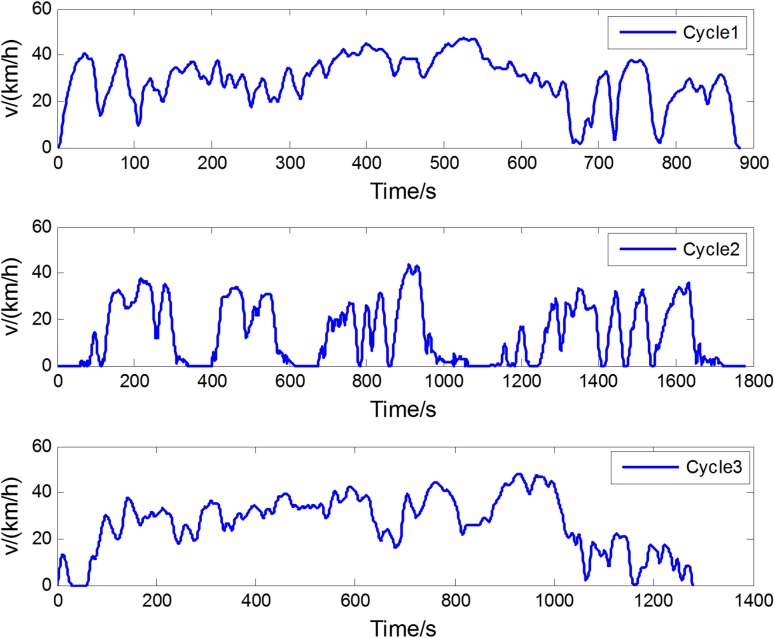
The three different driving schedules.

The three different driving schedules were adopted to validate the robustness of the RL-based real-time energy management strategy again, as shown in Fig 15. Table 5 lists the fuel consumption after *SOC*-correction. The real-time method, which utilizes the newest driving characteristics, gives superior fuel economy to the stationary one.

**Table 5:** Fuel consumption for three different driving schedules.

Cycle	Algorithms	Fuel consumption(g)	Relative increase	Final SOC
Cycle1	RL online	903.6	—	0.6958
	Stationary	960.4	6.3%	0.7012
Cycle2	RL online	1097.3	—	0.6923
	Stationary	1176.1	7.2%	0.6915
Cycle	RL online	1245.5	—	0.7021
	Stationary	1320.8	6.0%	0.6902

## 5. Conclusion

This paper proposes a real-time energy management strategy based on reinforcement learning for a hybrid electric tracked vehicle. A recursive algorithm for the transition probability matrix (TPM) is developed to make use of new statistical characteristics of online driving schedule. Based on the updating transition probability matrix (TPM), the control policy is calculated and updated at regular intervals to adapt to different driving conditions. A detailed facing-forward simulation model including the engine-generator model, battery model and vehicle dynamical model is built. In order to validate the effectiveness and adaptability of real-time control policy, the simulation for two driving schedules are operated. The results indicate that real-time energy management strategy has a superior fuel economy than stationary one, and is more effective in real-time control requirement.

## Supporting information

S1 Dataset The data for long naturalistic driving schedule. (MAT)

S2 Dataset The data for experimental driving schedule. (MAT)

S3 Dataset The data for validation driving schedule. (MAT)

S4 Dataset The data for another three driving schedules. (RAR)
